# Genetic features of red and green junglefowls and relationship with Indonesian native chickens Sumatera and Kedu *Hitam*

**DOI:** 10.1186/s12864-016-2652-z

**Published:** 2016-05-04

**Authors:** Maria Ulfah, Ryouka Kawahara-Miki, Achmad Farajalllah, Muladno Muladno, Ben Dorshorst, Alison Martin, Tomohiro Kono

**Affiliations:** Department of Animal Production and Technology, Faculty of Animal Science, Bogor Agricultural University (IPB), Bogor, West Java Indonesia; NODAI Genome Research Center, Tokyo University of Agriculture, Tokyo, Japan; Department of Biology, Faculty of Mathematics and Natural Science, Bogor Agricultural University (IPB), Bogor, West Java Indonesia; Department of Animal and Poultry Sciences, Virginia Tech, Blacksburg, VA USA; The Livestock Conservancy, Pittsboro, NC USA; Department of Bioscience, Tokyo University of Agriculture, Tokyo, Japan

**Keywords:** Red junglefowl, Green junglefowl, Indonesian native chicken, Whole-genome sequencing, SNP, Phylogeny, Mitochondrial DNA

## Abstract

**Background:**

More than 2,500 breeds of chicken are reared throughout the world as a source of eggs or meat and as pets. The primary ancestor of the present domestic chicken is widely believed to be the red junglefowl, although genetic contributions from other junglefowls cannot be excluded entirely. The reference genome for chicken was obtained from a red junglefowl, the genetic purity of which has been debated. There is, at present, insufficient data to resolve these interesting issues.

**Results:**

In this study, we performed whole-genome sequencing to compare various species and breeds of chicken, including wild red and green junglefowl, as well as the Indonesian native chickens Sumatera and Kedu *Hitam* and their respective descendants, the American Black Sumatra and Black Java. The data indicate that wild junglefowls have retained their genetic identity, but the Indonesian and American breeds have not. The Black Sumatra and Black Java are now closely related to each other, suggesting loss of genetic identity after export to the United States. In addition, the results indicate that the red junglefowl used as reference genome is more closely related to domestic chickens and apparently different from other wild red junglefowls.

**Conclusions:**

This study illuminates the genetic and phylogenetic relationships among these species. It provides a framework for genetic studies in wild junglefowls and native and domestic chicken breeds.

**Electronic supplementary material:**

The online version of this article (doi:10.1186/s12864-016-2652-z) contains supplementary material, which is available to authorized users.

## Background

After a long history of domestication and breeding dating back to 6,000 BC, more than 2,500 breeds of chicken are now raised worldwide as sources of eggs or meat and as pets [1]. The present domestic chicken has descended from the junglefowl, which belongs to the genus *Gallus*, order Galliformes. There are four living species, namely the red junglefowl (RJF) *Gallus gallus,* the green junglefowl (GJF) *G. varius*, the grey junglefowl *G. sonneratii,* and the Ceylon junglefowl *G. lafayettii*, which are found in India, Sri Lanka, Southeast Asia, and Indonesia. Of these, RJF is widely believed to be the primary ancestor of the present domestic chicken *Gallus gallus domesticus* [2–5]. Indeed, comparison with mitochondrial DNA from ancient bone samples revealed that domestication occurred at least 7,400 years ago from a common ancestral red junglefowl [6]. However, genetic contributions from the other three junglefowls cannot be totally excluded [3, 4, 7–11], and several reports suggest that the green [11] and grey [7] junglefowl may have bred with domestic chicken to produce hybrid birds. Unfortunately, there is insufficient data at present to resolve this interesting question, whereas studies in other species indicate that recruitment of wild animals into domesticated herds persisted over a long period after initial domestication [12].

Data from whole-genome sequencing significantly advanced the understanding of genetic diversity in chicken. Such data have been generated for RJF [13], in addition to single nucleotide polymorphisms (SNPs) in Chinese Silkie [14], broiler, and layer lines [15]. However, the RJF reference sequence may not represent pure, wild-type RJF [13], and the authors have acknowledged the possibility that the sequence is of a chicken interbred with domestic breeds. Therefore, it is necessary to obtain whole-genome sequencing data from a wild RJF, as well as from other junglefowl species.

**Fig. 1:**
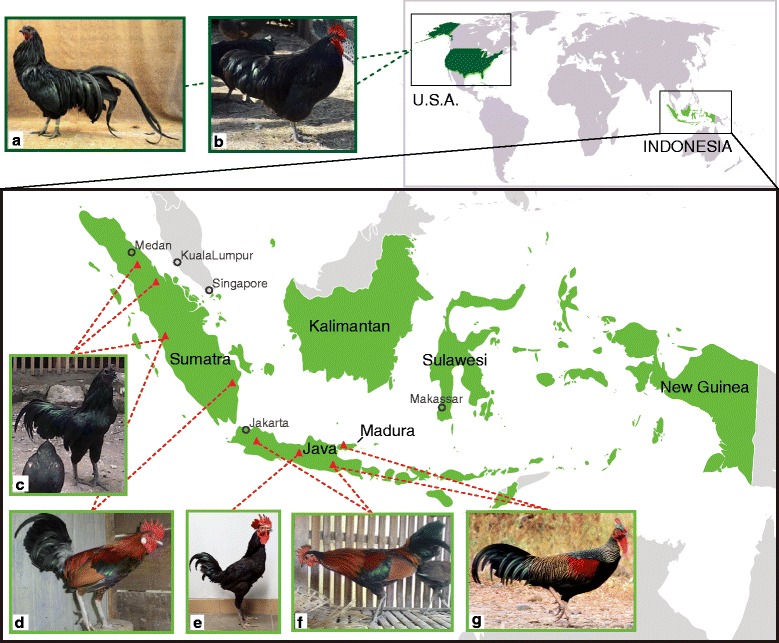
Chicken populations compared by whole-genome sequencing. **a**-**b**: American Black Sumatra (**a**) and Black Java (**c**). **c**: Sumatera chickens from three different geographical regions including Riau, north, and west Sumatra, Indonesia. **d**: Red junglefowl from west and south Sumatra, Indonesia. **e**: Kedu *Hitam* chickens from central Java, Indonesia. **f**: Red junglefowl from Java, Indonesia. **g**: Green junglefowl from Madura and east Java, Indonesia. Photos by Jeannette Beranger (**a**, **b**), Marka Hidayat (**c**), M. Fatchur Rohim (**d**), Maria Ulfah (**e**), Rahman Hidayat (**f**), and Reza Aulia Ahmadi (**g**). Map was retrieved from Wikimedia Commons [https://commons.wikimedia.org/wiki/File:Location_Southeast_Asia.svg]

Wild RJF is found on Sumatra, Java, and Madura islands, whereas wild GJF is found in Java, Madura, Bali, Lombok, Sumbawa, Flores, and Alor Islands in Indonesia [16]. A free-ranging variety of indigenous chicken, which is distinct from commercial breeds, is also found. At least 28 breeds of native chicken, totaling 290 million birds, are reared in Indonesia [17]. Of these, the Sumatera (English version is Sumatra) and Kedu *Hitam*, thought to have been bred for thousand years, are two of the oldest varieties and are used primarily for cockfighting and recently for ornamental purposes and as sources of eggs [18–20] (Fig. 1). These varieties are adapted to roughage diet and to the hot and humid climate. The Sumatera and Kedu *Hitam* chicken were brought to India, Europe, and America in the eighteenth century [21–25]. The Sumatera was admitted to the American Poultry Association Standard of Perfection in 1883 and named Black Sumatra (BS) in 1906 [26]. Kedu *Hitam* was crossed with other unknown breeds to develop Black Java (BJ), which was admitted to the American Standard in 1910 [26, 27].

Genomic comparison will help clarify the genetic contribution of RJF and GJF to Sumatera and Kedu *Hitam*, as well as to BS and BJ. Therefore, we compared, by whole-genome sequencing, RJF from Sumatra (RJFs) and Java (RJFj), GJF from Madura (GJFm) and Java (GJFj), the Indonesian Sumatera and Kedu *Hitam*, and the American BS and BJ (Fig. 1). Wild RJF and GJF from natural forests were used in the present study in order to compare their genomes with the reference chicken genome from RJF. These breeds were also compared with Japanese White Leghorn, White Plymouth Rock, and Rhode Island Red. The data illuminate the genetic and phylogenetic relationships among these species.

## Methods

### Samples

The study was conducted in compliance with the Bogor Agricultural University/IPB Animal Care and Use Committee (approved protocol number: 18-2014IPB). For sampling, the authors complied with the Convention on the Trade in Endangered Species of Wild Fauna and Flora and the IUCN Policy Statement on Research Involving Species a Risk of Extinction. Wild junglefowls were collected, and after blood sampling, immediately released back to their natural habitats. GJF was collected from a natural forest in the Tulungagung District, East Java (GJFj: 1 male, 1 female), and from karst areas in the Bangkalan District, Madura Island (GJFm: 2 males, 5 females). RJF was collected from a natural forest in the Madiun District, East Java, and in the Cipanas and Banten Districts, West Java (RJFj: 3 males). RJF was also collected from the Solok District, West Sumatra, and Ogan Komering Ilir District, South Sumatra (RJFs: 1 male, 1 female) (Fig. 1). The characteristics of pure junglefowl were determined based on a field guidebook for bird identification [28]. Samples of Indonesian native chickens, Sumatera (2 males, 3 females) and Kedu *Hitam* (5 males, 5 females), were obtained from farmers with unknown breeding practices. American BS and BJ were obtained from small-scale breeders. Inbred White Leghorn was provided by the Nagoya University Graduate School of Bioagricultural Science, Avian Bioscience Research Center, through the National Bio-Resource Project of the Ministry of Education, Culture, Sports, Science and Technology, Japan. Whole blood was collected from these birds, and genomic DNA was extracted by a combination of standard phenol/chloroform methods [29] and DNeasy Blood Kit (Qiagen, Valencia, CA, USA).

### Library preparation and DNA sequencing

Samples of genomic DNA (1μg) were fragmented to a median fragment size of 200 bp using a Covaris S2 Sample Preparation System (Covaris Inc., Woburn, MA, USA) and used to construct DNA libraries using NEBNext DNA Library Prep Reagent Set for Illumina (New England Biolabs, Ipswich, MA, USA). Library quality and quantity were evaluated using a 2100 Bioanalyzer (Agilent Technologies, Palo Alto, CA, USA) and a KAPA Library Quantification Kit (Kapabiosystems, Inc., Woburn, MA, USA). The libraries were used to generate clusters on an Illumina cBOT using a TrueSeq PE Cluster Kit v3-cBOT-HS (Illumina Inc., San Diego, CA, USA) and were sequenced using Hiseq 2500 (Illumina) with a TruSeq SBS Kit-HS (200 cycles; Illumina) as 100 bp paired-end reads. Sequence data are deposited in the DNA Data Bank Japan Sequence Read Archive (Accession No. DRA003951).

Sequence data from each bird were then mapped separately and compared with published data from domestic chickens White Plymouth Rock (Accession No. SRS524493) and Rhode Island Red (SRS524486).

### Mapping and SNP calling

Raw sequence reads from each bird were mapped separately to the galGal4 reference genome established in November 2011 using BWA program ver. 0.7.5a-r405 with default settings [30]. Read depth and coverage were then estimated based on the results. SNPs in uniquely mapped reads were identified in each bird by Samtools ver. 0.1.19-44428cd [31] according to criteria defined previously [15], with slight modifications in which SNPs were called at sites with read depth equal to or higher than the average and with 100 % polymorphic call rate. The SNPs were annotated using Refseq datasets. Insertions (sites that existed in the sequenced sample but not in the reference genome) and deletions (sites that existed in the reference genome but not in the sequenced sample) were identified in the same manner as SNPs.

Finally, gene ontology (GO) terms associated with genes containing non-synonymous SNPs, which were common in each breed, were extracted and summarized using Agbase [32, 33]. Enrichment analysis was performed using singular enrichment analysis as implemented by Agrigo [34]. Fisher’s exact test was used for statistical test. GO term of FDR < 0.05 was assumed significant [35].

### Phylogenetic analysis

Phylogenetic analysis was performed using SNP matrices for each individual. Prior to analysis, SNPs with sequence read depth below average were filtered out against the reference genome by an in-house perl script. SNPs with a minimum allele frequency of less than 5 % were also removed by Tassel ver. 5 [36]. The final SNP matrix of 14,554,492 sites, which included only variable sites, was used for further analysis. A pairwise genetic distance matrix between individuals was calculated based on the modified Euclidean distance [37], which was defined as D = 1-identity by state (IBS) similarity, where IBS is the probability that alleles derived at random from two individuals at identical loci are the same. For any two individuals, the probability of IBS was averaged using Tassel. An unrooted neighbor-joining (NJ) tree was constructed and robustness of the tree topology was assessed using 100 bootstrap replicates in PHYLIP ver. 3.695 [38].

Phylogenetic analysis was also performed using mitochondrial genome sequences. Sequences were first reconstructed by replacing corresponding sites in the reference sequence with SNPs found in other species. Mitochondrial genome sequence from the reference genome was not used in this analysis because the sample used for the reference genome sequence was different from that used for mitochondrial genome sequence. To construct phylogenetic trees, genes from mitochondrial genomes, excluding ND6 gene, were aligned separately using Clustal W implemented in the software package MEGA6 [39]. Gene alignments were then concatenated into protein-coding, tRNA, and rRNA genes. The protein-coding gene dataset was further partitioned into the 1st, 2nd, and 3rd position of the codons, and three datasets were constructed. The first dataset consisted of tRNA, rRNA, and the 1st, 2nd, and 3rd codons of protein-coding genes (123tr), while the second dataset consisted of tRNA, rRNA, and the 1st and 2nd positions of protein-coding genes (12tr). The final dataset contained tRNA, rRNA, the 1st and 2nd position, and RY coding for the 3rd position (123RYtr). Datasets were analyzed by partitioned Bayesian and partitioned maximum likelihood using MrBayes ver. 3.2.5 [40] and RAxML version 7.7.1 [41], respectively, with default settings.

## Results and discussion

### Sequencing and mapping

Various breeds of domestic chicken and wild junglefowl were analyzed by whole-genome sequencing using 2–10 birds per breed; > 15 Gb were obtained for each breed (Table 1). In all individuals except one GJFj bird, > 70 % of sequence reads were uniquely mapped to the reference genome (Additional file 1: Figure S1). About 5 % were mapped to multiple chromosomal locations, and up to 20 % were not mapped (Additional file 1: Figure S1). Coverage depth was averaged among individuals in each breed except in Rhode Island Red and White Plymouth Rock (in these breeds, the coverage depth was obtained from one sample). The average read depth was 15–80 in each breed (4–12 in each individual). In each breed except Rhode Island Red, > 90 % of the genome was covered by at least five sequence reads (Additional file 2: Figure S2). Sequence data from Rhode Island Red did not cover the whole genome, probably because of insufficient data in the published sequence. Chromosome 16 had relatively low coverage depth in all breeds, probably because the reference sequence was incomplete or mapping was difficult due to repetitive content in this chromosome, as previously noted [42]. Notably, GJFj and White Plymouth Rock had relatively sparse coverage at depth 10. Finally, coverage of chromosome W varied depending on the sex of the samples.

**Table 1:** Summary of sequenced data and average genetic distance in each breed

Project	Sample #	Yield (Mbases)	% PF^*a^	# Reads	% of > = Q30 bases (PF)	Mean quality score (PF)	Genetic distance^*b^	1-IBS^*c^
Green junglefowl in Java	2	15,930	100	159,310,248	96.36	37.46	0.495	0.361
Green junglefowl in Madura	7	57,444	100	574,445,124	96.04	37.33	0.454 ± 0.01^*d^	0.339 ± 0.008
Red junglefowl in Sumatra	2	25,807	100	258,067,414	94.72	36.91	0.125	0.114
Red junglefowl in Java	3	24,355	100	243,545,708	95.43	37.16	0.162 ± 0.003	0.145 ± 0.002
Sumatera	5	25,432	100	254,326,066	95.13	37.04	0.164 ± 0.005	0.146 ± 0.004
Black Sumatra	10	40,056	100	400,575,800	95.1	37.05	0.171 ± 0.012	0.152 ± 0.009
Kedu *Hitam*	10	76,000	100	759,981,010	93.87	36.61	0.142 ± 0.010	0.128 ± 0.008
Black Java	10	86,169	100	861,690,196	91.02	35.60	0.131 ± 0.021	0.119 ± 0.017
Total/Average	49	351,193	100	3,511,941,566	94.62	36.86	0.161 ± 0.071	0.189 ± 0.105

^*a^% PF means that the percentage of total number of passing filter reads per sequenced reads

^*b^K2P genetic distance was calculated by Phylip

^*c^IBS (Identity by state) was calculated by Tassel

^*d^Standard deviation

### SNP analysis

**Fig. 2:**
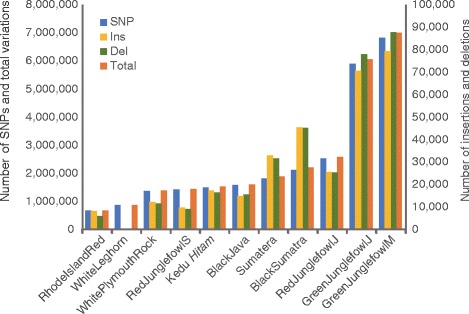
Histogram of SNPs (blue), insertions (yellow), deletions (green), and total (red). The number of SNPs and indels were averaged from individuals in each breed, except for Rhode Island Red and White Plymouth Rock. RedJunglefowlS, red junglefowl from Sumatra; RedJunglefowlJ, red junglefowl from Java; GreenJunglefowlM, green junglefowl from Madura; GreenJunglefowlJ, green junglefowl from Java

The average number of SNPs in each breed was calculated and compared among the breeds (Fig. 2). GJFm (*n* = 699 × 10^4^) and GJFj (*n* = 604 × 10^4^) had more SNPs than did the others (*n* = 68–258 × 10^4^), suggesting that these junglefowls were more distant to the reference RJF (Fig. 2). Unexpectedly, wild RJFj (*n* = 258 × 10^4^) had many more SNPs than domestic chickens, even though the reference sequence was a RJF, whereas wild RJFs had the similar number of SNPs to domestic chicken (Rhode Island Red, White Leghorn, and White Plymouth Rock). The domestic chickens Rhode Island Red (*n* = 68 × 10^4^) and White Leghorn (*n* = 86 × 10^4^) had the lowest number of SNPs against the reference (Fig. 2). White Plymouth Rock, RJFs, Kedu *Hitam*, and BJ possessed a similar number of SNPs (*n* = 136–156 × 10^4^), whereas Sumatera (*n* = 182 × 10^4^) and BS (*n* = 222 × 10^4^) had relatively more (Fig. 2). Similar results were obtained based on insertions and deletions (indels), although these variations were very few in White Leghorn (Fig. 2).

Pairwise genetic distance between the breeds was estimated using the SNP data (Table 1, Additional file 3: Figure S3). The highest genetic distance was observed between GJFm and GJFj (Table 1), indicating high genetic variation among individuals from the GJF breeds compared to other varieties.

The average numbers of SNPs and indels in each breed were similarly distributed in the genome (Additional file 4: Figure S4). Approximately 79–80 % were intergenic, while 16–18 % were genic; most of the genic variations were in introns. However, the number of non-synonymous and synonymous variations in exons was different between SNPs and indels. In particular, thousands of SNPs were present in exons, of which 579–7,564 were found to cause non-synonymous amino acid changes. In contrast, several tens of indels at the most caused amino acid gain or loss (Additional file 4: Figure S4).

To detect additional breed specific variations, we performed GO analysis focusing on genes containing non-synonymous SNP, which is common within each breed. Kedu *Hitam* was excluded from further analysis because of a too small number of genes containing non-synonymous SNPs (6 genes; Additional file 5: Table S1). GO terms associated with the non-synonymous SNP containing genes were obtained and summarized via GO slim terms (Additional file 6: Figure S5). Several differences in the percentage of GO slim terms to total GO slim annotation were found in Sumatera and BS breeds (Additional file 6: Figure S5), albeit these differences were not statistically significant.

Furthermore, GO enrichment analysis against the chicken reference genome demonstrated that RJFj and GJFj showed two GO terms, which were significantly enriched in each breed (Additional file 5: Table S1). Further functional analysis is necessary to examine the molecular mechanisms that are represented by these significantly enriched GO terms. Nevertheless, the significant enrichment of GO terms reflects the distant relationship between these breeds and the reference genome.

### Phylogenetic analysis

**Fig. 3:**
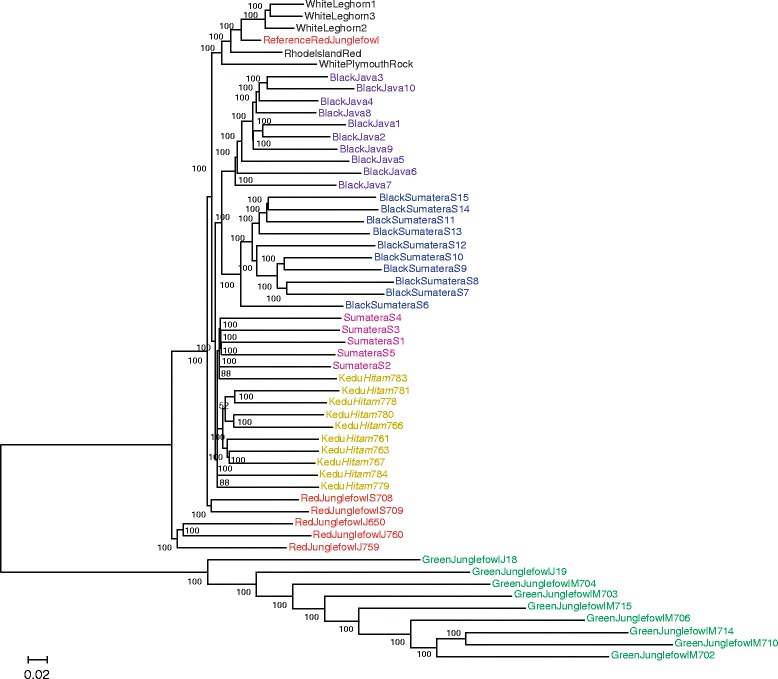
Neighbor-joining tree based on 14,554,492 SNPs. Bootstrap values from 100 replicates are shown on internal branches. Abbreviations are defined in Fig. 2. Numbers after the name of a breed are the sample numbers. Colors on labels are colored by breeds. Red junglefowl are in red, green junglefowl are in green, Black Sumatra in blue, Black Java in purple, Sumatera in pink, Kedu *Hitam* in yellow, and Rhode Island Red, White Plymouth Rock, and White Leghorn are in gray

Relationships among the individuals were further investigated by phylogenetic analysis of 14,554,492 SNPs, and NJ tree was constructed with 100 bootstrap iterations (Fig. 3). Most of internal branches received 100 % bootstrap support, except for several internal clades that were part of a cluster containing Kedu *Hitam* and Sumatera.

GJFm and GJFj formed a cluster, showing that GJF is phylogenetically distinct, with negligible genetic contribution to other varieties. In addition, GJFm birds had long branches more than other species did, indicating high genetic variation within the species. In contrast, wild RJF from Sumatra (RJFs) and Java (RJFj), as well as the reference RJF, did not form a monophyletic group, indicating diverging genetic backgrounds. RJFj formed a monophyletic group that was sister to the GJF group, which in turn formed a monophyletic group with RJFs. Surprisingly, the reference RJF formed a monophyletic group with domestic chickens (Rhode Island Red, White Plymouth Rock, and White Leghorn), which relationship supports the hypothesis that the chicken used for reference genome sequence had interbred with domestic breeds [13]. The separation of RJFj, RJFs, and the reference RJF suggests that these breeds have differentiated by founder effects, genetic drift, or incomplete lineage sorting as their ancestral species separated on different islands. Further analysis using RJF from other islands and countries will clarify the detailed genetic structure of RJF populations in Indonesia and neighbor countries and help elucidate the detailed scenario for domestication of chicken. The monophyly of three birds of White Leghorn, which were from the inbred closed colony, was confirmed. One Kedu *Hitam* bird formed a monophyletic group with Sumatera chicken, whereas the remaining Kedu *Hitam* birds formed a separate but neighboring clade. Monophyly of Kedu *Hitam* and Sumatera indicated close relationship among the Indonesian native chicken breeds. BS and BJ also formed separate groups but shared a sister-group relationship, suggesting a close relationship between the two American breeds. However, these two breeds did not show a close relationship with other domestic chicken breeds. Finally, the analysis did not support a distinct relationship between Sumatera and BS and between Kedu *Hitam* and BJ.

As the analysis of all SNPs in this study did not resolve the problems of incomplete lineage sorting and the anomaly zone, the resultant phylogeny, particularly the short internodes, might lead to different conclusions. Future multi-species coalescent studies would refine the phylogeny of these species based on the SNP matrix.

**Fig. 4:**
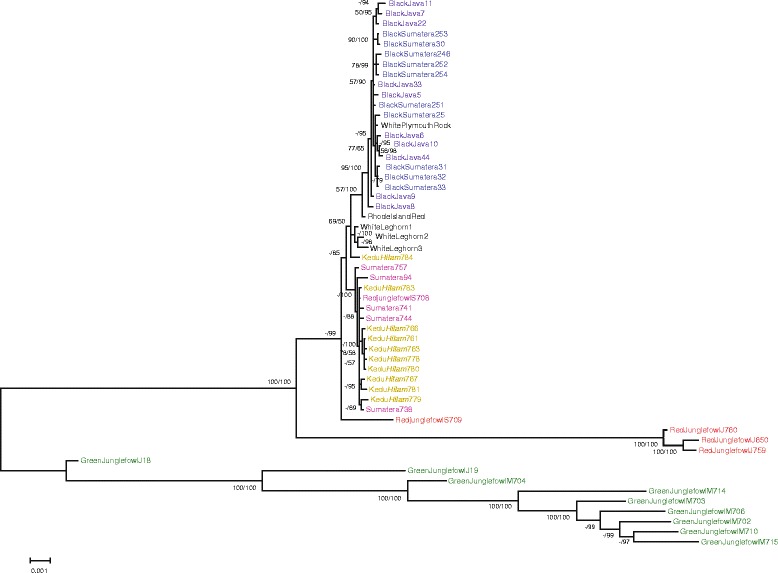
Bayesian consensus tree based on mitochondrial genome. Numbers beside internal branches indicate bootstrap values >50 % from 100 replicates (left) and Bayesian posterior probabilities (right). (-) indicates a node not recovered in the maximum-likelihood analysis or bootstrap values <50 %. Abbreviations are defined in Fig. 2. Numbers after the name breed are the sample numbers. Colors on labels are defined in Fig. 3

Phylogenetic relationships were also reconstructed using mitochondrial genomes, resulting in six phylogenies based on three datasets and two methods. The topology reconstructed by Bayes method from a dataset containing rRNA, tRNA, and 1st, 2nd, and 3rd codon of the protein-coding genes (123tr) is shown in Fig. 4. This topology seems reasonable, as it reflects the monophyly of three birds of White Leghorn, which were from a closed colony. However, the support values in the mitochondrial tree are generally low for both the posterior probabilities of the Bayesian analysis and the bootstrap values of the maximum-likelihood analysis (Fig. 4). GJFm and GJFj formed a monophyletic group that was a sister clade to RJFj. RJF birds did not form a monophyletic group, suggesting diverse genetic origins, as we have noted in the analysis of SNPs. Similarly, Sumatera and Kedu *Hitam* groups were not closely related to BS and BJ, respectively. In particular, the American BS and BJ formed a monophyletic, poorly supported group with White Plymouth Rock and other domestic chicken breeds (Rhode Island Red and White Leghorn), implying that BS and BJ have been crossed with other chickens in the United States.

The difference between the topology inferred from the SNPs and that inferred from the mitochondrial genome sequences reflects the different amount of sequence data or different inheritance pattern. Nevertheless, the poor support in the mitochondrial genome phylogeny questions whether the inferred topology reflects the underlying biology.

## Conclusion

In this study, we evaluated the genetic contribution of RJF and GJF to Indonesian (Sumatera and Kedu *Hitam*), American (BS and BJ), and domestic chicken (Rhode Island Red, White Plymouth Rock, and White Leghorn) breeds. The results show that genetic identity is conserved in GJF, and that this species has made little genetic contribution to the domestic chicken (Figs. 3 and 4). In contrast, RJF breeds are genetically heterogeneous, perhaps reflecting the original genetic diversity of this species or a history of cross breeding between wild and domestic species. In particular, the Indonesian Sumatera and American BS and likewise Kedu *Hitam* and BJ do not cluster together as breed history would have suggested, presumably because of crossbreeding in the United States.

Thus this study illuminates the genetic and phylogenetic relationships among wild junglefowls and native and domestic chicken breeds and provides a framework for genetic studies in these species. The results suggest that conserved morphological similarity does not necessarily reflect the conserved genetic background, demonstrating the importance of and difficulty in conserving genetic diversity of wild and indigenous chicken species. Therefore, the whole genome sequencing provides a great tool for addressing these challenges.

### Ethics

Ethical approval was obtained from Animal Care and Used Committee of the Bogor Agricultural University/IPB (protocol number: 18-2014IPB) and research procedures to animal were complied with the Convention on the Trade in Endangered Species of Wild Fauna and Flora and the IUCN Policy Statement on Research Involving Species at Risk of Extinction.

### Consent to publish

Not applicable.

### Availability of data

Sequence data were deposited in the DNA Data Bank Japan Sequence Read Archive (Accession No. DRA003951).

## Additional files

Additional file 1: Figure S1. Mapping. Data are percentage of sequence reads mapped to unique regions in the reference genome (blue), to multiple regions (red), and not mapped (green). Abbreviations are defined in Figure 2. Numbers after the breed name are sample numbers. (PDF 89 kb) Additional file 2: Figure S2. Genome coverage averaged in each breed. Data are percentage of regions covered by ≥ 1 (red), ≥ 3 (light blue), ≥ 5 (blue), and ≥ 10 (green) sequence reads in each chromosome. Abbreviations are defined in Fig. 2. (PDF 106 kb) Additional file 3: Figure S3. Genetic distance (K2P, upper triangle; 1-IBS, lower triangle) in each breed. Abbreviations are defined in Fig. 2. (PDF 538 kb) Additional file 4: Figure S4. Distribution of SNPs (blue), deletions (green), and insertions (yellow) in the genome. The number in each region is averaged in each breed. Upstream and downstream variations are within 5 kb of genes. Abbreviations are defined in Fig. 2. (PDF 210 kb) Additional file 5: Table S1. Summary of non-synonymous SNP containing genes fixed in each breeds and associated GO terms. (PDF 48 kb) Additional file 6: Figure S5. Distribution of gene ontology (GO) slim terms associated with non-synonymous single nucleotide polymorphism (SNP) containing genes, which are common in each breed. GO terms were categorized by biological process, cellular component, and molecular function, as indicated. Abbreviations are defined in Fig. 2. (PDF 89 kb)

## Abbreviations

BJ: Black Java
BS: Black Sumatra
GJF: green junglefowl
GJFj: green junglefowl from Java
GJFm: green junglefowl from Madura
GO: gene ontology
Indels: insertions and deletions
MEXT: ministry of education, culture, sports, science and technology
RJF: red junglefowl
RJFj: red junglefowl from Java
RJFs: red junglefowl from Sumatra
SNPs: single nucleotide polymorphisms
