# Supplementary figures and images for: Genetic features of red and green junglefowls and relationship with Indonesian native chickens Sumatera and Kedu Hitam
Source: BMC Genomics. 2016 May 4;17:320. doi: 10.1186/s12864-016-2652-z (PMC4855759; doi:10.1186/s12864-016-2652-z)

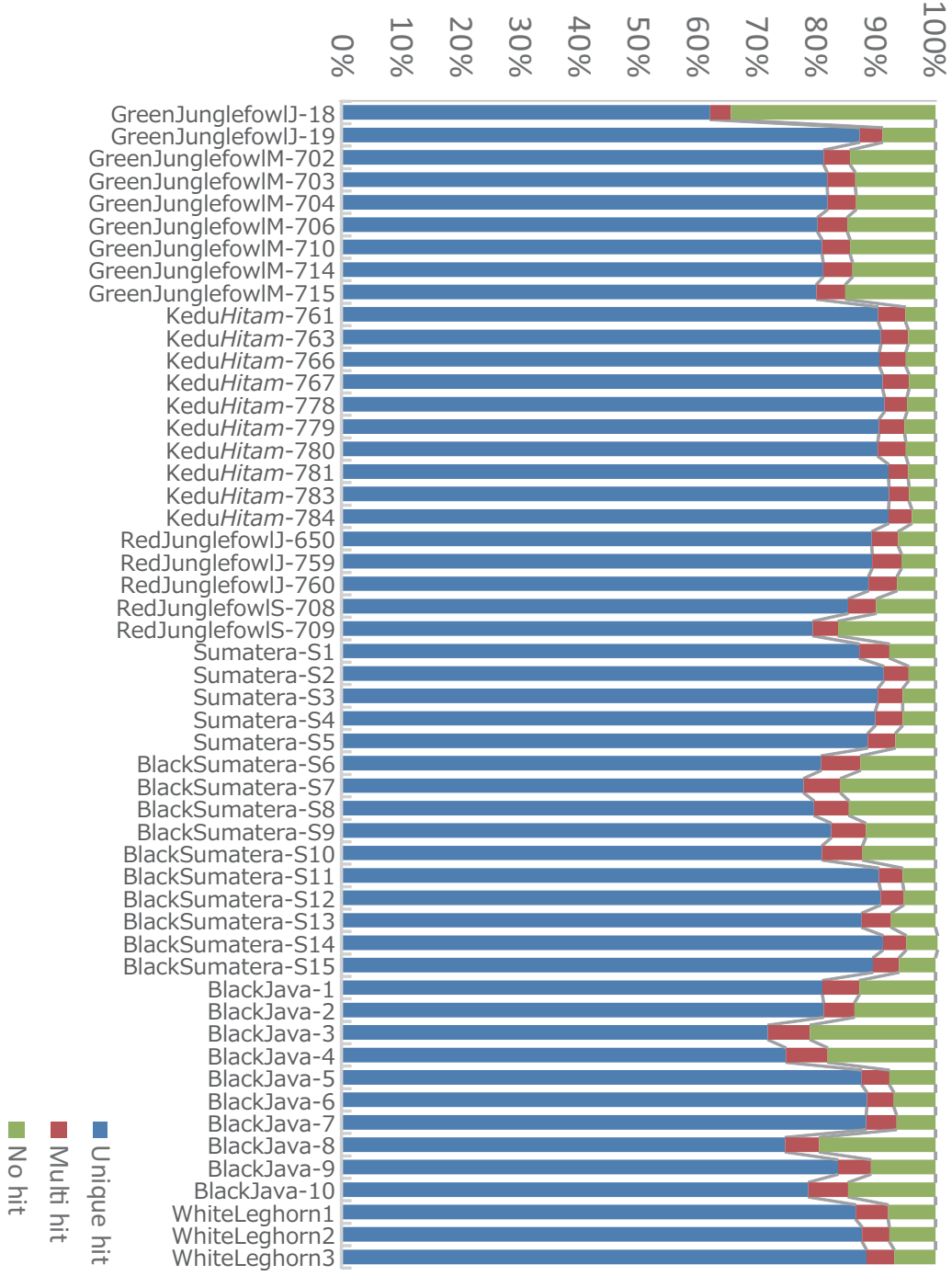

Supplement: Additional file 1: Figure S1. — Mapping. Data are percentage of sequence reads mapped to unique regions in the reference genome (blue), to multiple regions (red), and not mapped (green). Abbreviations are defined in Figure 2. Numbers after the breed name are sample numbers. (PDF 89 kb) [file 12864_2016_2652_MOESM1_ESM.pdf]

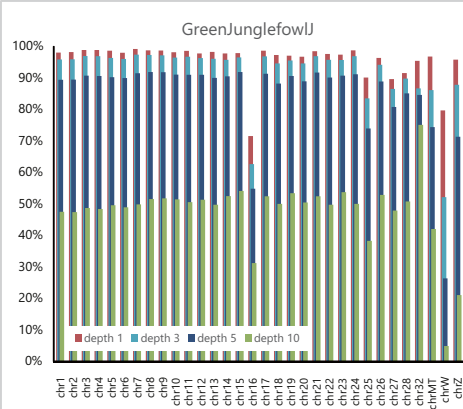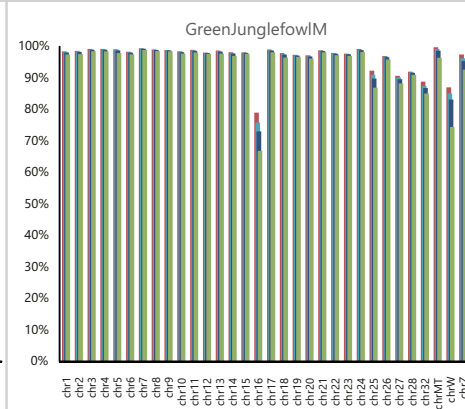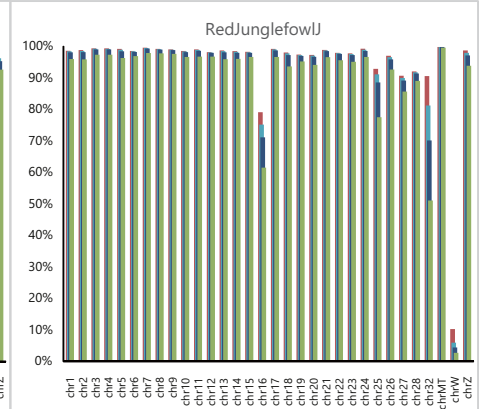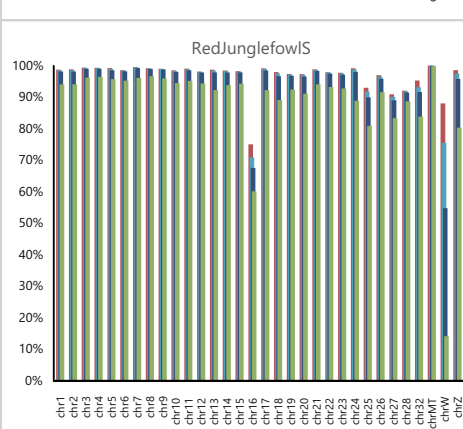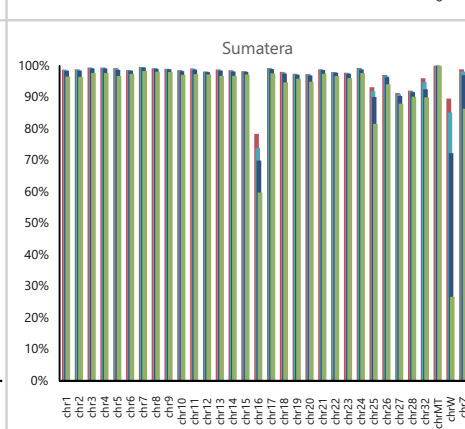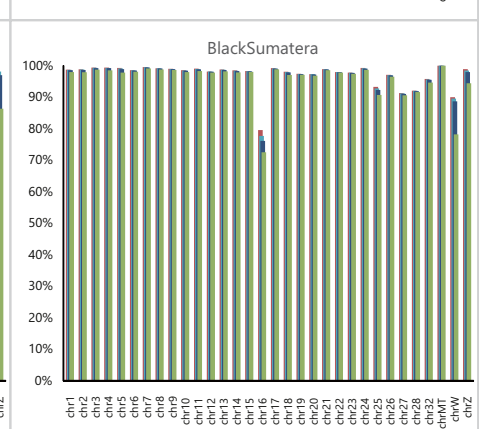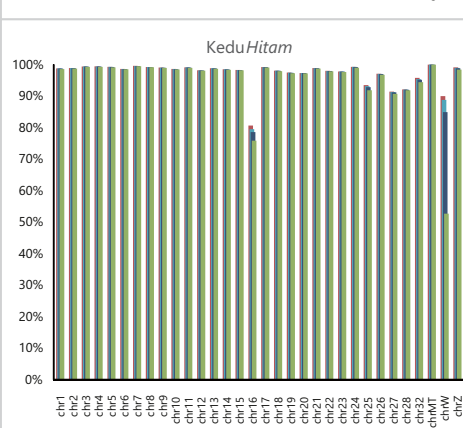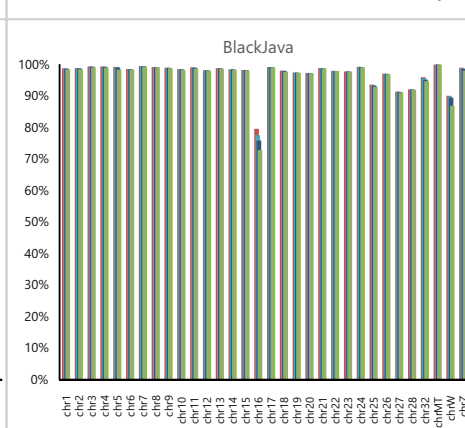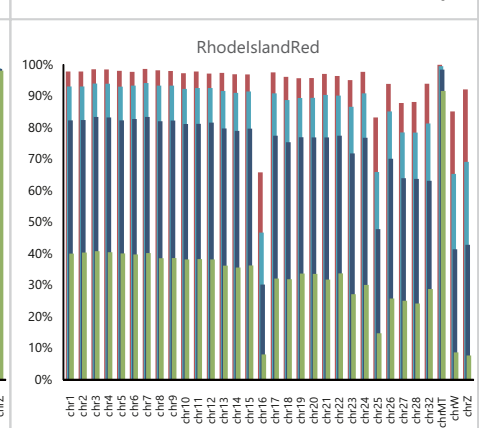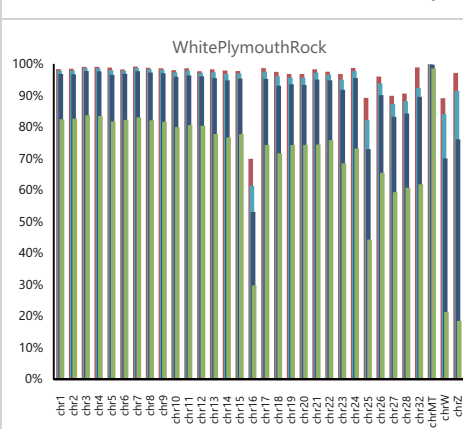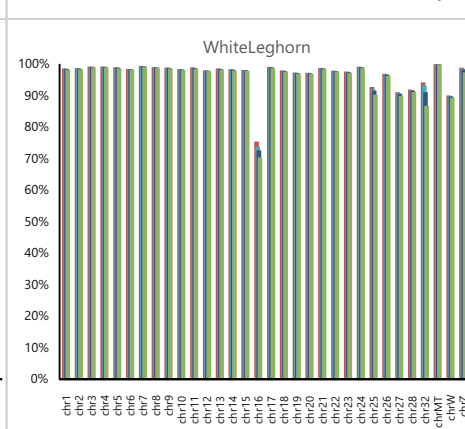

Supplement: Additional file 2: Figure S2. — Genome coverage averaged in each breed. Data are percentage of regions covered by ≥ 1 (red), ≥ 3 (light blue), ≥ 5 (blue), and ≥ 10 (green) sequence reads in each chromosome. Abbreviations are defined in Fig. 2. (PDF 106 kb) [file 12864_2016_2652_MOESM2_ESM.pdf]

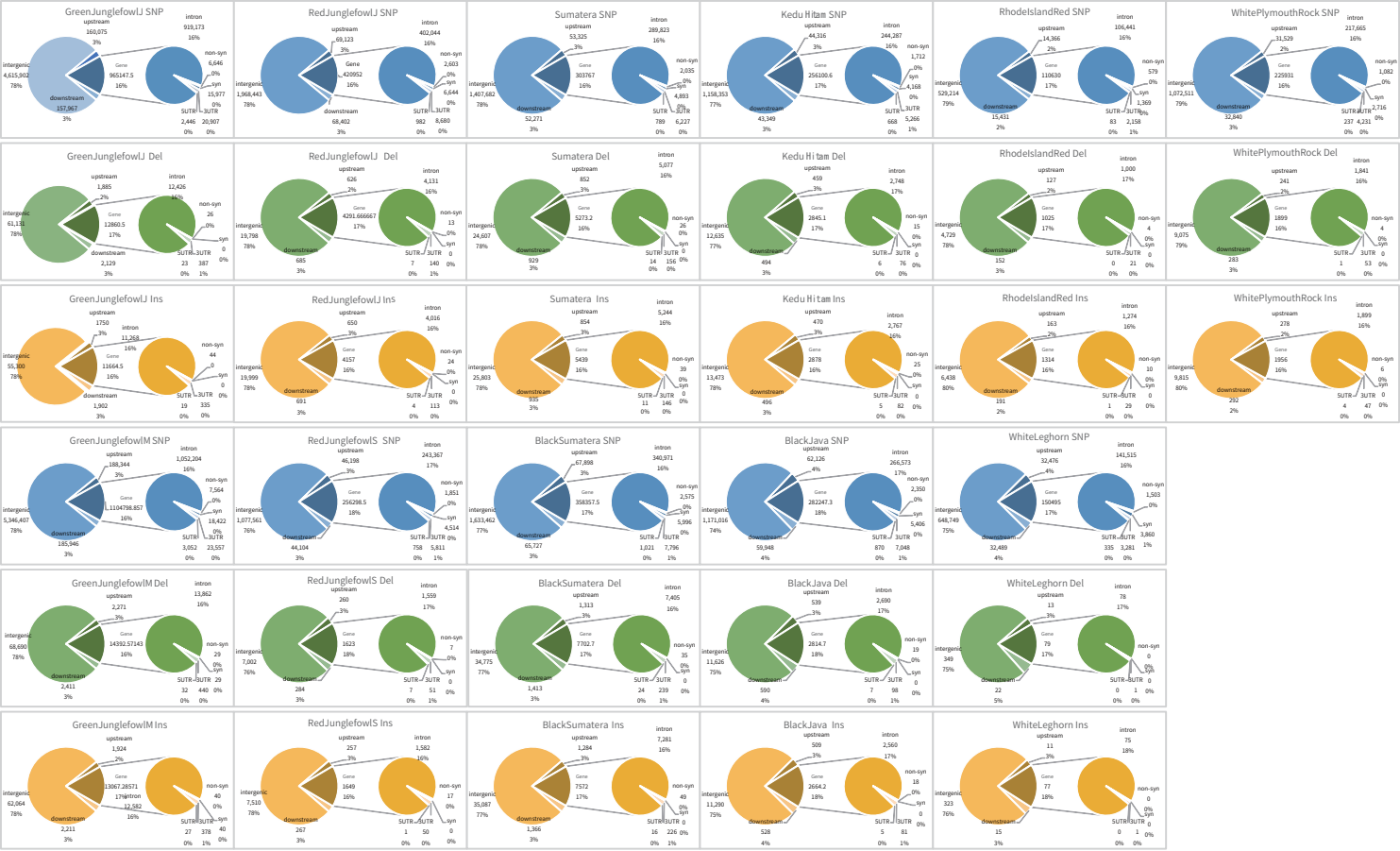

Supplement: Additional file 4: Figure S4. — Distribution of SNPs (blue), deletions (green), and insertions (yellow) in the genome. The number in each region is averaged in each breed. Upstream and downstream variations are within 5 kb of genes. Abbreviations are defined in Fig. 2. (PDF 210 kb) [file 12864_2016_2652_MOESM4_ESM.pdf]

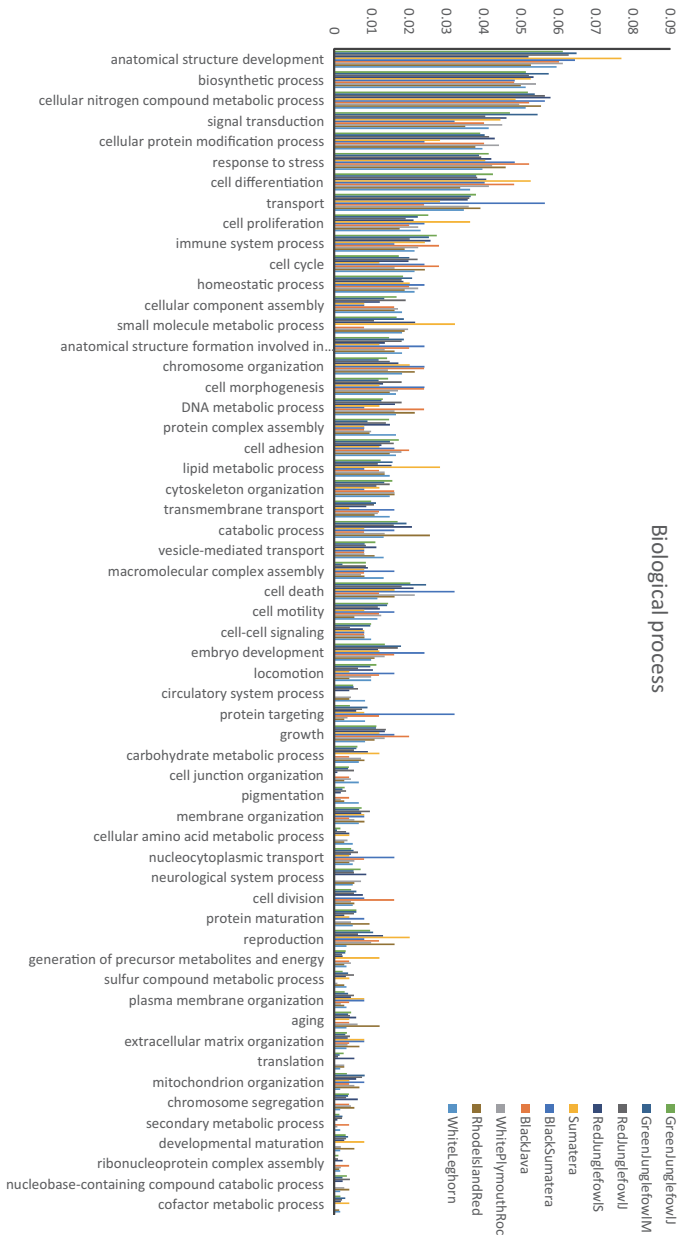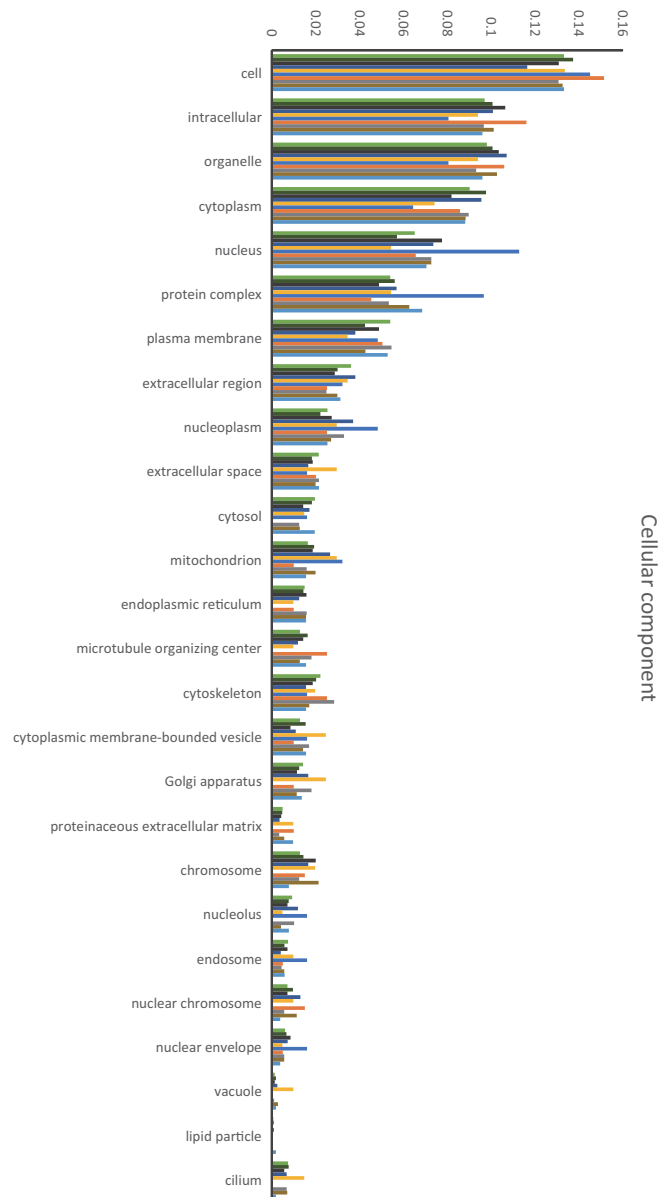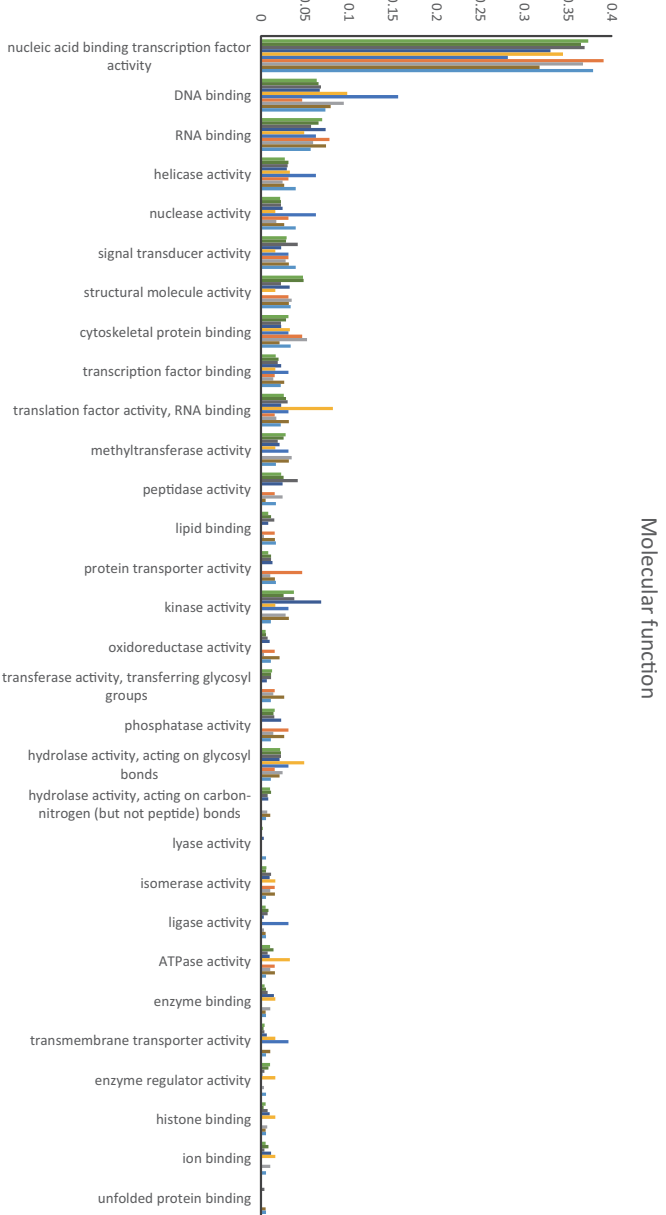

Supplement: Additional file 6: Figure S5. — Distribution of gene ontology (GO) slim terms associated with non-synonymous single nucleotide polymorphism (SNP) containing genes, which are common in each breed. GO terms were categorized by biological process, cellular component, and molecular function, as indicated. Abbreviations are defined in Fig. 2. (PDF 89 kb) [file 12864_2016_2652_MOESM6_ESM.pdf]
