# Supplementary material for: Genetic features of red and green junglefowls and relationship with Indonesian native chickens Sumatera and Kedu Hitam
Source: BMC Genomics. 2016 May 4;17:320. doi: 10.1186/s12864-016-2652-z (PMC4855759; doi:10.1186/s12864-016-2652-z)
Supplement: Additional file 3: Figure S3. — Genetic distance (K2P, upper triangle; 1-IBS, lower triangle) in each breed. Abbreviations are defined in Fig. 2. (PDF 538 kb) [file 12864_2016_2652_MOESM3_ESM.pdf]

GreenJunglefowlJ

	GJFj18	GJFj19
GJFj18		0.495
GJFj19	0.361	

GreenJunglefowlM

	702	703	704	706	710	714	715
702		0.455	0.461	0.441	0.438	0.451	0.450
703	0.339		0.459	0.446	0.463	0.476	0.450
704	0.343	0.342		0.458	0.477	0.481	0.453
706	0.332	0.334	0.341		0.438	0.452	0.446
710	0.330	0.344	0.351	0.329		0.421	0.462
714	0.337	0.351	0.353	0.338	0.320		0.467
715	0.336	0.337	0.338	0.334	0.343	0.346	

RedJunglefowlJ

	708	709
708		0.125
709	0.114	

RedJunglefowlS

	650	759	760
650		0.159	0.164
759	0.143		0.163
760	0.146	0.146	

Sumatera

	S1	S2	S3	S4	S5
S1		0.163	0.169	0.171	0.165
S2	0.146		0.161	0.161	0.155
S3	0.151	0.144		0.167	0.162
S4	0.152	0.144	0.149		0.164
S5	0.147	0.140	0.145	0.147	

BlackSumatera

	BS_S6	BS_S7	BS_S8	BS_S9	BS_S10	BS_S11	BS_S12	BS_S13	BS_S14	BS_S15
BS_S6		0.175	0.181	0.169	0.164	0.161	0.170	0.168	0.169	0.166
BS_S7	0.155		0.154	0.161	0.156	0.186	0.183	0.184	0.186	0.183
BS_S8	0.160	0.138		0.159	0.158	0.187	0.181	0.186	0.186	0.183
BS_S9	0.151	0.144	0.142		0.137	0.179	0.174	0.178	0.177	0.177
BS_S10	0.147	0.140	0.142	0.125		0.176	0.177	0.174	0.178	0.177
BS_S11	0.144	0.164	0.164	0.158	0.156		0.169	0.159	0.150	0.147
BS_S12	0.151	0.161	0.160	0.154	0.157	0.151		0.179	0.175	0.176
BS_S13	0.149	0.162	0.163	0.158	0.154	0.143	0.158		0.165	0.158
BS_S14	0.150	0.163	0.164	0.156	0.157	0.135	0.155	0.147		0.158
BS_S15	0.148	0.161	0.161	0.157	0.156	0.133	0.156	0.142	0.141	

Kedu *Hitam*

	761	763	766	767	780	781	783	784	778	779
761		0.118	0.142	0.121	0.128	0.134	0.132	0.147	0.147	0.131
763	0.108		0.139	0.122	0.137	0.137	0.133	0.150	0.149	0.134
766	0.128	0.126		0.142	0.146	0.149	0.130	0.154	0.161	0.148
767	0.111	0.112	0.129		0.135	0.136	0.136	0.143	0.149	0.135
780	0.117	0.124	0.132	0.123		0.145	0.137	0.137	0.157	0.143
781	0.122	0.125	0.135	0.124	0.131		0.141	0.154	0.150	0.136
783	0.121	0.121	0.119	0.124	0.125	0.127		0.146	0.155	0.141
784	0.133	0.135	0.139	0.129	0.125	0.138	0.132		0.166	0.155
778	0.133	0.134	0.144	0.134	0.141	0.135	0.139	0.148		0.148
779	0.120	0.122	0.133	0.123	0.129	0.124	0.128	0.139	0.134	

BlackJava

	BJ1	BJ2	BJ3	BJ4	BJ5	BJ6	BJ7	BJ8	BJ9	BJ10
BJ1		0.108	0.125	0.112	0.153	0.164	0.138	0.110	0.125	0.139
BJ2	0.100		0.115	0.103	0.141	0.154	0.130	0.103	0.117	0.134
BJ3	0.115	0.106		0.093	0.131	0.151	0.138	0.098	0.121	0.110
BJ4	0.103	0.096	0.087		0.134	0.148	0.126	0.084	0.105	0.115
BJ5	0.138	0.128	0.120	0.122		0.170	0.155	0.136	0.147	0.131
BJ6	0.146	0.139	0.136	0.133	0.151		0.162	0.149	0.158	0.169
BJ7	0.126	0.119	0.125	0.116	0.139	0.145		0.122	0.133	0.159
BJ8	0.102	0.096	0.092	0.079	0.124	0.135	0.112		0.105	0.122
BJ9	0.115	0.108	0.111	0.098	0.133	0.141	0.121	0.098		0.137
BJ10	0.126	0.122	0.102	0.106	0.119	0.150	0.143	0.112	0.125	

Upper triangle: genetic distance (Kimura 2 parameter)

Lowere triangle:1-IBS
