# Supplementary material for: Genetic features of red and green junglefowls and relationship with Indonesian native chickens Sumatera and Kedu Hitam
Source: BMC Genomics. 2016 May 4;17:320. doi: 10.1186/s12864-016-2652-z (PMC4855759; doi:10.1186/s12864-016-2652-z)
Supplement: Additional file 5: Table S1. — Summary of non-synonymous SNP containing genes fixed in each breeds and associated GO terms. (PDF 48 kb) [file 12864_2016_2652_MOESM5_ESM.pdf]

Table S1. Summary of non-synonymous SNP containing genes fixed in each breeds and associated GO terms.

Project	Number of non-sysnonymous SNP containing genes	Number of Go terms associated to common non-synonymous SNPs containing genes	Significantly enriched GO terms [*a]
Red junglefowl in Sumatera	775	2,512	-
Red junglefowl in Java	654	1,332	ATP-dependent helicase activity (GO: 0008026)<br>purine NTP-dependent helicase activity (GO: 0070035)
Green junglefowl in Madura	1,007	1,853	-
Green junglefowl in Java	3,879	3,628	oxidation reduction (GO: 0055114)<br>lipid localization (GO: 0010876)
Sumatra	158	389	-
Kedu *Hitam*	6	19	-
Black Sumatera	73	351	-
Black Java	213	564	-
Rhode Island Red	593	1,101	-
White Plymouth Rock	1,092	1,541	-
White Leghorn	698	982	-

[*a] FDR < 0.05
